# Gouty Panniculitis with Ulcerations in a Patient with Multiple Organ Dysfunctions

**DOI:** 10.1155/2014/320940

**Published:** 2014-06-15

**Authors:** Lu Wang, Crystal Rose, Paul Mellen, George Branam, Maria M. Picken

**Affiliations:** ^1^Department of Pathology and Laboratory Medicine, Loyola University Medical Center, 2160 S 1st Avenue, Maywood, IL 60153, USA; ^2^Department of Pathology, Ball Memorial Hospital, Indiana University Health, 2401 W University Avenue, Muncie, IN 47303, USA

## Abstract

Gouty panniculitis is a rare manifestation of gout. Clinically, it is characterized by indurated subcutaneous nodules in nonjoint areas. Pathologically, typical characteristic gouty tophi can be seen in subcutaneous tissue. It is postulated that gouty panniculitis develops as a consequence of uric acid accumulation in the body and localized inflammatory changes in subcutaneous tissue. We report a case of a 46-year-old man with 20-year history of gout, who developed multiple subcutaneous nodules over the abdomen and right groin/thigh area over a 2-year period. After a recent episode of congestive heart failure and acute renal failure, the nodules increased in size and the overlying skin became erythematous and ulcerated. Pathologic examination demonstrated typical tophi in the dermis and subcutaneous tissue. A review of the literature yielded fifteen similar cases that had been previously reported. We conclude that gouty panniculitis may be a manifestation of undertreated gout and may be exacerbated by the deterioration of other systemic functions.

## 1. Introduction

Gouty panniculitis is a rare manifestation of gout. It is characterized clinically by indurated, erythematous, or ulcerated subcutaneous nodules. Histologic confirmation is achieved by identification of tophaceous crystal deposition in the lobular subcutaneous tissue [1, 2]. The clinical presence of indurated subcutaneous plaques may precede, or appear subsequently to the articular clinical expression of tophaceous gout [1]. We report a case of gouty panniculitis manifesting as multiple subcutaneous nodules in different locations with and without ulceration. The recent literature related to this disease entity is also reviewed.

## 2. Case Report

A 46-year-old male with a 20-year history of gout presented with nontender nodules in the subcutaneous tissue of the abdomen, groin, and right thigh. The nodules had been present for two years; however, over the past three weeks, some of the nodules became erythematous and eventually ulcerated. In addition, the patient had recently been admitted for congestive heart failure and acute renal failure 3.5 weeks prior to presentation. His gout had never been treated. Furthermore, the patient's medical history was complicated by type II diabetes and morbid obesity with a BMI of 40.6.

**Figure 1:**
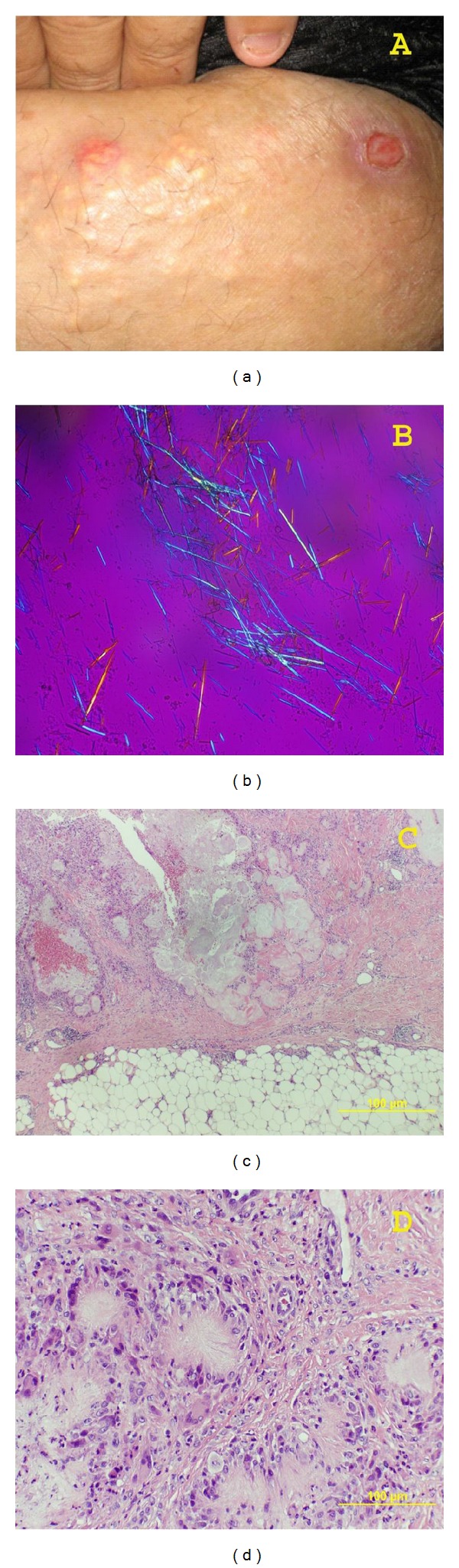
Skin and subcutaneous lesion. (a) Ulcer and erythematous nodule in right groin area, 1 to 1.5 cm; (b) negative birefringence of urate crystal on fresh tissue touch imprint slide (400x); (c) and (d) subcutaneous lesion: low power picture shows subcutaneous tissue erosion with pools of pale staining or slightly eosinophilic amorphous/feathery material surrounded by collagen fibers and chronic inflammatory cells (c, 40x); high power picture shows feathery eosinophilic crystalline material converging in the center and surrounded by palisading histiocytes and multinucleated giant cells (d, 200x).

Physical examination revealed multiple, whitish subcutaneous nodules and 4 skin ulcers in the lower half of the abdomen, right groin, and right thigh. All ulcers measured between 1 and 3 cm; fibrinous, necrotic, and chalky whitish material covered poorly formed granulation tissue. The nonulcerated pale, white nodules with focal erythema could be palpated subcutaneously and averaged from 1.0 to 1.5 cm in diameter (Figure 1(a)). Clinically, there was also significant swelling of the right elbow and the MCP joints on the patient's right hand.

Results of laboratory tests from the patient's most recent admission revealed the following: uric acid 14.3 mg/dL (reference range 4–7 mg/dL), blood glucose 255 mg/dL (reference range 70–99 mg/dL), BUN 47 mg/dL (reference range 5–20 mg/dL), creatinine 1.61 mg/dL (reference range 0.8–1.4 mg/dL), and estimated GFR 46 mL/min/1.73 m^2^ (reference range ≥59). His uric acid levels were 10.8 mg/dL and 11.2 mg/dL at the time of surgical removal of the ulcerated nodules, 3 and 4 weeks later. At that time also, his renal function and blood glucose levels were still abnormal: BUN 38 mg/dL, creatinine 1.65 mg/dL, GFR 45 mL/min/1.73 m^2^, and blood glucose 147 mg/dL.

The patient underwent 3 excisional biopsies, including 2 from an abdominal skin lesion and 1 from the right thigh. All 3 specimens were submitted separately, over a 3-week period, for histological evaluation. Sectioning of the specimens revealed multifocal areas filled with white, chalky material. Touch imprint slides were prepared before tissue fixation. Microscopic examination under polarized light showed multiple needle-shaped crystals displaying negative birefringence (Figure 1(b)). Permanent histology slides showed pools of pale and slightly eosinophilic amorphous as well as feathery material in the dermis and subcutaneous tissue (Figures 1(c) and 1(d)). The lesions demonstrated different stages of development. The smaller lesions had a central area of feathery crystalline material surrounded by palisading histiocytes and multinucleated giant cells. The larger lesions consisted of disorganized, laminated material surrounded by collagen fibers and chronic inflammation. The lesions eroded outward to the skin surface leading to ulceration. Based on the clinical, laboratory, and histologic findings, a diagnosis of multifocal gouty tophi was made.

## 3. Discussion

Gout has become increasingly common in the Western world, such that the lifetime risk of acquisition is now approximately 1-2% [3, 4]. Tophi are a common clinical manifestation of gout. They contain collections of monosodium urate crystals and are commonly located in the vicinity of joints of the elbows, hands, and feet. They lead to joint destruction and chronic (long-term and continuous) joint pain and stiffness. Gouty panniculitis is characterized by inflammation of the subcutaneous fat and manifests itself clinically as nodular (lumpy) lesions on the legs and trunk, which ulcerate and ooze fluid containing monosodium urate crystals [5]. In contrast to tophi, panniculitis has been reported, thus far, only in a very small percentage of patients with gout. Thus, Webershock [2] reported one case in 2010 and reviewed 8 prior reported cases from 1977 to 2007 [2, 6–9]. Recently, however, Ochoa et al. [1] reported an additional 6 cases from a single institution and concluded that panniculitis should be considered as one of the principal clinical manifestations of gout. These authors also raised the question of whether this particular manifestation has hitherto been under reported.

The pathogenesis of gouty panniculitis is currently not well understood. Some predisposing factors may enhance the deposition of monosodium urate crystals in the subcutaneous tissue. As expected, hyperuricemia is present in the vast majority of patients diagnosed with this condition [7]. A high metabolic rate, leading to increased production of uric acid, may lead to an oversaturation of monosodium urate with joint deposition and deposition in the lobular subcutaneous tissue [1]. In addition, preexisting subcutaneous tissue damage is known to increase the risk of subcutaneous panniculitis. Additional factors may include venous stasis, varicosities, or chronic edema related to cardiac failure and elevated serum amylase or lipase [2, 7]. Elevated serum amylase and lipase, in patients with renal failure and end stage renal disease, may be due to decreased renal clearance of these enzymes [10, 11]. Hence, chronic renal insufficiency associated with hypertensive nephropathy or other medical conditions represents a potential risk factor for gouty panniculitis [1].

Ochoa et al. postulated that gouty panniculitis could be caused by localized inflammatory changes in the lobular subcutaneous tissue. This inflammation may be triggered or perpetuated by blood supply disruption caused by monosodium urate crystals, consequently resulting in microtrauma to the terminal capillary walls and adipose tissues. This hypoxia then renders the subcutaneous tissues vulnerable to further injury [1].

More recently, uric acid has been shown to activate the NLRP3 (NOD (nucleotide-binding oligomerization domain) like receptors, pyrin domain 3) inflammasome, which plays a key role in innate immunity. In lipopolysaccharide (LPS) stimulated mice, monosodium urate (as well as calcium pyrophosphate dihydrate) crystals have been shown to cause an increase in caspase-1 activation and the secretion of IL-1*β*. However, mice deficient for inflammasome components were defective in crystal-induced IL-1*β* secretion [12]. Moreover, in a peritoneal murine model of gout, monosodium urate crystal-recruited monocytes differentiate into proinflammatory M1-like macrophages producing more IL-1 along with other cytokines and chemokines [13]. These experimental findings support the concept that the innate immune system may play a critical role in the triggering of crystal-induced acute inflammation. Consonant with these findings, IL-1 inhibitors appear to be beneficial in the treatment of gout.

Our patient had a long history of untreated gout. Over two years, he progressively developed subcutaneous nodules over his abdomen, groin, and right thigh area. His blood uric acid levels ranged between 10.8 mg/dL and 14.3 mg/dL during his recent hospitalization. The subcutaneous nodules became erythematous and ulcerated soon after the development of congestive heart failure and acute renal failure. His renal failure was a likely consequence of his cardiac insufficiency, the latter providing low blood perfusion of the kidney. In addition, this patient was diabetic and may have had underlying diabetic nephropathy. This in turn could have made the kidney more susceptible to impaired perfusion due to the acute onset of heart failure. The deterioration of kidney function and the use of the loop diuretic furosemide may have accelerated the accumulation of uric acid in the patient's body [14], ultimately exacerbating his gouty panniculitis. Furthermore, the severe hyperuricemia might have led to further deterioration of renal function. Histologically, the newly developed small lesions, which were surrounded by palisading histiocytes, may be indicative of the active phase of his panniculitis. The patient's other medical conditions, which contributed to gout development, included type II diabetes [15] and morbid obesity [16].

The differential diagnosis of gouty panniculitis encompasses a wide range of disorders including sclerema neonatorum, subcutaneous fat necrosis, pancreatic panniculitis, poststeroid panniculitis, factitial panniculitis, hemorrhagic panniculitis, and many others [1, 2, 7]. Although gouty panniculitis can often demonstrate a granulomatous reaction, the detection of feathery crystalline material, although rare, is helpful. Since monosodium urate crystals can be dissolved during paraffin fixation and routine hematoxylin and eosin (H&E) staining, the feathery structures seen on permanent histology slides actually represent empty spaces previously occupied by the needle-shaped crystals of monosodium urate. Thus, the direct detection of crystals using polarized light requires either touch imprint slides prepared from fresh tissue or the use of ethanol fixation during tissue processing.

All gouty panniculitis cases (18 in total, including the current case) are reviewed. The average age of onset is 47.2 years old. In general, gouty panniculitis is a late clinical manifestation of chronic gout (average 17 years). However, the skin lesions may also be manifested before the onset of classic gout [1]. Gouty panniculitis appears more likely to develop in male patients, with a male to female ratio of 7 : 1. Hyperuricemia (mean 9.8 mg/dL) plays an important role in the development gouty panniculitis. Some patients, who have a normal uric acid level at diagnosis, may have experienced long-term hyperuricemia at an earlier time. Among all 18 cases, abnormal renal function was noted in 12 (66.7%) patients [1, 6, 8, 9, 17, 18]. Due to the lack of complete metabolic panel (CMP) data, renal function in the remaining 6 cases could not be assessed [2, 7, 19]. Also, elevation of erythrocyte sedimentation rate and C-reactive protein have been noted in some cases [2, 7, 8].

With regard to gouty panniculitis, although no specific treatment for this condition is currently available, systemic anti-inflammatory therapy, including corticosteroids, can often rapidly ameliorate the symptoms [2, 7]. The nonhealing ulcerated lesions can also be surgically resected. Our patient was treated with NSAIDs (naproxen) and prednisone with a fair response.

## 4. Conclusion

Although gouty panniculitis is an uncommon symptom of gout, the development of nodules in subcutaneous tissue should alert the clinician to the need for a more careful monitoring of the patient. Uncontrolled hyperuricemia can lead to devastating dermatopathologic consequences. Skin manifestations can be an indicator of undertreated or untreated gout, as well as the decompensation of other systemic conditions such as renal dysfunction. Further systemic workup or close clinical monitoring should be considered in such patients.
